# Individual patient data meta-analysis of trials investigating the effectiveness of intra-articular glucocorticoid injections in patients with knee or hip osteoarthritis: an OA Trial Bank protocol for a systematic review

**DOI:** 10.1186/2046-4053-2-54

**Published:** 2013-07-05

**Authors:** Marienke van Middelkoop, Krysia S Dziedzic, Michael Doherty, Weiya Zhang, Johannes W Bijlsma, Timothy E McAlindon, Stefan L Lohmander, Sita MA Bierma-Zeinstra

**Affiliations:** 1 Department of General Practice, Erasmus MC Medical University, PO Box 2040, 3000, CA, Rotterdam, the Netherlands; 2 Arthritis Research UK Primary Care Centre, Primary Care Sciences, Keele University, Staffordshire, ST5 5BG, UK; 3 Department of Rheumatology, University of Nottingham, Nottingham, UK; 4 Epidemiology and Methodological, University of Nottingham, Nottingham City Hospital, Hucknall Road, Nottingham NG5 1PB, UK; 5 Department of Rheumatology & Clinical Immunology, University Medical Center Utrecht, PO Box 85500, 3508, GA, Utrecht, the Netherlands; 6 Department of Medicine, Tufts University, 136 Harrison Avenue, MA 02111, Boston, USA; 7 Department of Orthopaedics, Clinical Sciences, Lund University Hospital, 22185, Lund, Sweden; 8 Institute of Sports Science and Clinical Biomechanics and Department of Orthopaedics and Traumatology, University of Southern Denmark, Syddansk Universitet Øster Farimagsgade 5 A, 2, DK-1399, Copenhagen, Denmark

**Keywords:** Osteoarthritis, Individual patient data, Corticosteroid injection

## Abstract

**Background:**

Based on small to moderate effect sizes for the wide range of symptomatic treatments in osteoarthritis (OA), and on the heterogeneity of OA patients, treatment guidelines for OA have stressed the need for research on clinical predictors of response to different treatments. A meta-analysis to quantify the effect modified by the predictors using individual patient data (IPD) is suggested. The initiative to collect and analyze IPD in OA research is commenced by the OA Trial Bank. The study aims are therefore: to evaluate the efficacy of intra-articular glucocorticoids for knee or hip OA in specific subgroups of patients with severe pain and (mild) inflammatory signs, over both short-term and long-term follow-up, using IPD from existing studies; to reach consensus on the rules for cooperation in a consortium; and to develop and explore the methodological issues of meta-analysis with individual OA patient data.

**Methods/Design:**

For the current IPD analysis we will collect and synthesize IPD from randomized trials studying the effect of intra-articular glucocorticoid injections in patients with hip or knee OA. Subgroup analyses will be performed for the primary outcome of pain at both short-term and long-term follow-up, in the subgroups of patients with and without severe pain and with and without inflammatory signs.

**Discussion:**

This study protocol includes the first study of the OA Trial Bank, an international collaboration that initiates meta-analyses on predefined subgroups of OA patients from existing literature. This approach ensures a widely supported initiative and is therefore likely to be successful in data collection of existing trials. The collaboration developed (that is, the OA Trial Bank) may also lead to future IPD analyses on subgroups of patients with several intervention strategies applied in OA patients.

## Background

Based on small to moderate effect sizes of the wide range of symptomatic treatments in osteoarthritis (OA), and on the heterogeneity of OA patients, treatment guidelines for OA have stressed the need for research on clinical predictors of response to different treatments [1,2]. Identifying responsive subgroups is not simple and it is essential to use the correct methodology in order to prevent patients being erroneously deprived of beneficial treatments, or erroneously assumed to have an (improved) effect from such treatment. Subgroup-specific trials are obvious for the different OA joint groups (for example, hand, hip, knee or foot) and for treatment specifically aimed at certain OA subgroups such as osteotomy for varus knee OA [3]. However, to design trials for every identified subgroup with the available treatments would be resource consuming and unrealistic.

*Post hoc* analyses within individual trials are frequently applied to identify subgroups with respect to effect of treatment. However, these methods of analysis introduce a high risk of type I and type II errors and are therefore unreliable [4,5]. A methodologically robust method is to test for subgroup–treatment interaction effects [3]. This method carries a much smaller risk of false-positive results, but large sample sizes are necessary to detect the effect modification; that is, interaction between subgroup variable and treatment. A meta-analysis for quantifying interaction effects using individual patient data (IPD) might overcome the power problem in individual trials and is therefore considered to be the gold standard for subgroup analysis [6]. This method requires re-analysis of IPD made available by the authors of several trials. In a meta-analysis using IPD, in which the data from several trials are pooled, the interaction effects between subgroups and treatment can be reliably assessed and potential confounders can be adjusted for [6].

The initiative to collect and analyze IPD in OA research is commenced by the OA Trial Bank. The OA Trial Bank will bring together data from individuals with OA recruited to different clinical trials from different countries around the world to form a databank. Potential subgroups of patients for different interventions in OA patients will be predefined by the OA Trial Bank and will be analyzed with IPD.

The first IPD analysis will be performed on the efficacy of intra-articular (i.a.) glucocorticoid injection. For patients who are unresponsive to non-invasive treatments or oral NSAIDs, i.a. glucocorticoid injections can provide an opportunity to treat OA in the knee or hip. An i.a. corticosteroid injection is recommended for OA patients particularly with signs of local inflammation with joint effusion [2,7-10]. The Cochrane systematic review on the effectiveness of i.a. corticosteroid injection in knee OA found some evidence for the efficacy of i.a. corticosteroid injections compared with i.a. placebo for pain and patient global assessment at 1 week post injection, with evidence also for continuing efficacy at 2 and 3 weeks post injection [9]. There are, however, suggestions that there are subgroups of patients who do, and do not, respond to i.a. glucocorticoid injections. A study by Jones and Doherty concluded that the response to the treatment was not confined to those patients with clinical evidence of inflammation [11]. Another study did find a greater improvement for pain among patients with clinical evidence of joint effusion [12]. Since previous systematic reviews have not considered IPD, interaction effects between inflammatory signs and glucocorticoid injections are largely unclear. The primary aim of this study is therefore to evaluate the efficacy of i.a. glucocorticoids for knee or hip OA in specific subgroups of patients with severe pain and (mild) inflammatory signs, over both short-term and long-term follow-up, using IPD from existing trials. In addition to the primary aim, we hope to reach consensus on the rules for cooperation in a consortium and to develop and explore the methodological issues of meta-analysis with IPD.

## Methods/Design

We will carry out an IPD meta-analysis of randomized trials studying the effectiveness of i.a. glucocorticoid injections in patients with hip or knee OA. The protocol of this review is not registered in the PROSPERO database.

### Study selection

The following inclusion criteria will be applied for studies to be included in the OA Trial Bank for the current study purpose.

#### *Type of studies*

All randomized controlled trials, including crossover studies, evaluating one or more i.a. glucocorticoid preparations in patients with OA of the knee or hip will be included. There will be no language restrictions.

#### *Participants*

Participants are males and/or females with a diagnosis of OA of the knee or hip according to published American College of Rheumatology classification criteria [13,14], or on the basis of detailed clinical and/or radiographic information.

Studies including a subgroup of knee or hip OA patients are also included, as long as IPD are collected.

#### *Types of interventions*

All i.a. glucocorticoid preparations used for treatment of OA of the knee or hip in humans were compared with control treatments – including placebo (saline, vehicle; that is, inactive medium in which drugs can be administered), i.a. hyaluronan/hylan, other doses of i.a. glucocorticoids, usual conservative treatment (pain medication and/or exercise therapy) – or were compared with different types of injection procedures of glucocorticoids.

#### *Types of baseline assessments*

First, important confounders of severity of pain, age, gender and body mass index should at least have been assessed at baseline. Also, if available, signs of inflammation should be assessed at baseline, either by physical examination (warmth, effusion) or by additional testing (ultrasound, magnetic resonance imaging, biopsy, serum C-reactive protein/erythrocyte sedimentation rate).

#### *Types of outcomes*

The minimum criterion for inclusion of trials in the systematic review is adequate reporting of pain. Information regarding other outcome measures from the OMERACT III core set, such as physical function, and patient global assessment will be analyzed when feasible [15]. The primary outcome measure is pain severity at short-term (up to 3 weeks) follow-up. There will be no restrictions regarding duration of follow-up.

### Subgroup analyses

Subgroup analyses will be performed for the primary outcome of pain at both short-term and long-term follow-up, in the subgroups of patients with and without severe pain and with and without inflammatory signs. In addition, secondary explorative analyses will be performed for other subgroups of patients, dependent on the amount of data available.

### Identification of eligible studies

The following databases will be searched from 1995 (based on availability of datasets and authors) until 19 June 2012 for randomized controlled trials of i.a. glucocorticoid versus control treatment for OA of the knee or hip: the Cochrane Central Register of Controlled Trials (CENTRAL), MEDLINE (PubMed), EMBASE, Web of Science, Scopus, Cinahl, Pedro and the controlled trial registers. The search strategy is derived from the Cochrane review on the efficacy of i.a. corticosteroid injections in knee OA [9]. The detailed search syntax is presented in Additional file 1: Appendix 1. Reference lists will be hand searched for further identification of published work. Additional potential ongoing studies are also search by means of the horizon scanning documents from the UK and the clinical trial registries.

Two review authors (MvM, SMAB-Z) will independently first select citations based on titles and abstracts. Next, full articles are obtained for those citations thought to fulfill the inclusion criteria and screened by the two review authors independently. A third review author will be consulted if consensus is not reached.

### Data collection and transfer

All corresponding authors of eligible trials will be approached and asked to cooperate in this project. When we cannot reach the corresponding authors, the institutes in which the trials have been performed will be contacted. All data deliverers (that is, the research institutes who own the data) will be asked to sign the data delivery license agreement, including items on input data, obligations, ownership of data, terms, authorship and publications. The coordinator of the OA Trial Bank will visit the institutes of the data deliverers once; to collect the data and to sign the license agreement. Datasets will be accepted in any kind of electronic format (for example, SPSS, Stata, SAS, Excel) or in paper form, provided that variables and categories are adequately labeled within the dataset or with a separate codebook. The original data collection files collected by the coordinator will be kept in their original version and will be saved on a secured server at the Erasmus MC Medical University in Rotterdam. To ensure the quality of the data, they will be independently checked for data-entry mistakes and consistency, and the sum of the individual patient results received will be compared with the published summary results from the primary studies. In the case of differences, authors will be contacted and discrepancies should be resolved after discussion.

To ensure accurate pooling of data, all items will be consistently derived from the original databases by the coordinator of the OA Trial Bank and will consequently be recoded if necessary. All anonymous data will be transferred to a secured database at the Erasmus MC Medical University in Rotterdam. The dataset will not be used for any other research apart from that described in the license agreement.

### Risk of bias assessment

The methodological quality of all included clinical trials in the OA Trial Bank will be assessed using the 12 criteria recommended by the Cochrane Collaboration and will be evaluated independently by two researchers (Table 1 and Additional file 1: Appendix 2). The criteria are scored as ‘yes’ (low risk of bias), ‘no’ (high risk of bias) or ‘unclear’. Any disagreements between the review authors are resolved by discussion, including input from a third review author. A study with a low risk of bias is defined as fulfilling six or more of the criteria items, which is supported by empirical evidence.

**Table 1:** Sources of risk of bias

	**Item**^**a**^	**Judgment**
A) Sequence generation		
	1. Was the method of randomization adequate?	Yes / No / Unsure
B) Allocation concealment		
	2. Was the treatment allocation concealed?	Yes / No / Unsure
C) Was knowledge of the allocated interventions adequately prevented during the study?		
	3. Was the patient blinded to the intervention?	Yes / No / Unsure
	4. Was the care provider blinded to the intervention?	Yes / No / Unsure
	5. Was the outcome assessor blinded to the intervention?	Yes / No / Unsure
D) Incomplete outcome data		
	6. Were incomplete outcome data adequately addressed?	
	E) Other sources of potential bias	
	7. Were all randomized participants analyzed in the group to which they were allocated?	Yes / No / Unsure
	8. Were the groups similar at baseline regarding the most important prognostic indicators?	Yes / No / Unsure
	9. Were co-interventions avoided or similar?	Yes / No / Unsure
	10. Was the compliance acceptable in all groups?	Yes / No / Unsure
	11. Was the timing of the outcome assessment similar in all groups?	Yes / No / Unsure

^a^Criteria for a judgment of ‘yes’ for the sources of risk of bias: see Additional file 1: Appendix 2.

### Data extraction

From the published reports, details of the trial design, interventions and comparator groups will be obtained. Data on factors used to stratify the study sample will also be collected (Table 2).

**Table 2:** Input data from individual randomized trials

	**Type of data**
**Baseline**	
***At least available input data:***	
Trial number	Created by OA Trial Bank
Patient ID	Random number
Date of randomization	Date
Age	Continuous
Gender	Dichotomous (male/female)
Body mass index (or weight and length)	Continuous
Type of osteoarthritis	Dichotomous (hip/knee)
Type of intervention	String variable
Pain severity (VAS, NRS, WOMAC, other)	Continuous
***Collected for current pilot study:***	
Signs of inflammation	Depends on measurement method
- physical examination	
- imaging (that is, sonography, magnetic resonance imaging)	
- additional testing (ESR, CRP, inflammatory markers)	
***Collected when available:***	
Osteoarthritis characteristics:	Depends on measurement method
- American College of Rheumatology criteria	
- Severity	
- Radiographic information	
- Kellgren and Lawrence score	
Duration of complaints	Continuous or dichotomous
Physical functioning (WOMAC/KOOS/KSS/…)	Continuous
Patient global assessment	Continuous or dichotomous
**Outcome measures at follow-up**	
***At least available input data:***	
Pain severity (VAS, NRS, WOMAC, other)	Continuous
***Collected when available:***	
Physical functioning (WOMAC/KOOS/KSS/…)	Continuous
Patient global assessment	Continuous or dichotomous

CRP, C-reactive protein; ESR, erythrocyte sedimentation rate; KOOS, Knee injury and Osteoarthritis Outcome Score ; KSS, Knee Society Score ; NRS, Numerical Rating Scale ;VAS, Visual Analogue Scale ; WOMAC, Western Ontario and McMaster Universities Osteoarthritis Index.

Data obtained from the original databases include patient characteristics (age, gender, body mass index), disease-specific characteristics (American College of Rheumatology criteria, radiographic information, signs of inflammation, duration of complaints), study characteristics (trial number, types of interventions, doses) and outcome measures at both baseline and follow-up measurements (pain, function and global perceived recovery). All randomized patients with a database record will be entered in the pooled database. All individual trials will be allocated an individual random trial number. Patients lost to follow-up or excluded in published per-protocol analyses will be included in the database of the OA Trial Bank.

### Data analyses

Overall effects between the different comparative treatments and within these comparisons will be estimated in the pooled IPD. Descriptive comparisons between studies will be conducted to assess between-study differences. We assume the data to be missing at random, and therefore observed patient characteristics will be used to impute missing data (potential covariates and outcomes) by means of multiple imputation [16,17]. Missing data will be imputed within each original study, before data of the individual studies are pooled. Treatment effects will be analyzed using a random-effects model. The heterogeneity between the separate trials will be tested with *I*^2^[18]. An additional analysis will be performed by excluding the trials causing heterogeneity in order to reach an *I*^2^ index <50.

The analyses will be adjusted for variables used in stratified randomization procedures when necessary.

The primary outcome is pain severity at short-term follow-up. When pain severity is measured on different scales, we will standardize these pain scores in order to pool the data. Secondary outcomes include pain severity assessed at other follow-up durations, physical functioning and global assessment [15].

The subgroup factor will, based on consensus, be standardized to: yes or no severe pain; and yes or no signs of mild inflammation and yes or no signs of moderate to severe inflammation. In addition, separate pooled analysis, if possible, will take place for the different assessments to define inflammation, and for hip and knee OA.

### One-step approach

A multilevel regression analysis will be applied to estimate the magnitude of the effects in the different subgroups with the individuals nested within each study (permits to include both study-level and patient-level covariates in the same model). Subgroup analyses will be performed by including a single covariate in the regression model to indicate the study in order to adjust for possible residual confounding by study differences. Other covariates define possible confounders that can be distributed unequally over the treatments in subgroups. Age, gender and body mass index will be included as a minimum, but if possible duration of complaints, radiographic severity and educational level will also be included.

To assess potential subgroup effects, a random-effects linear regression model will be used to calculate interaction effects. The model will include the dependent variable (that is, pain intensity at follow-up (0 to 100 point scale)), independent variables (that is, treatment (glucocorticoid injection or control)), the effect modifier (severe pain (yes or no) and signs of inflammation (yes or no)), and an interaction term (pain × treatment or inflammation × treatment).

The pooled subgroup effect of glucocorticoid injections will be estimated according to a mean difference (for continuous outcomes) and odds ratio (for binary outcomes) and their 95% confidence intervals, based on the intention-to-treat principle. Interaction effects with *P* <0.05 will be considered statistically significant.

### The OA Trial Bank

**Figure 1:**
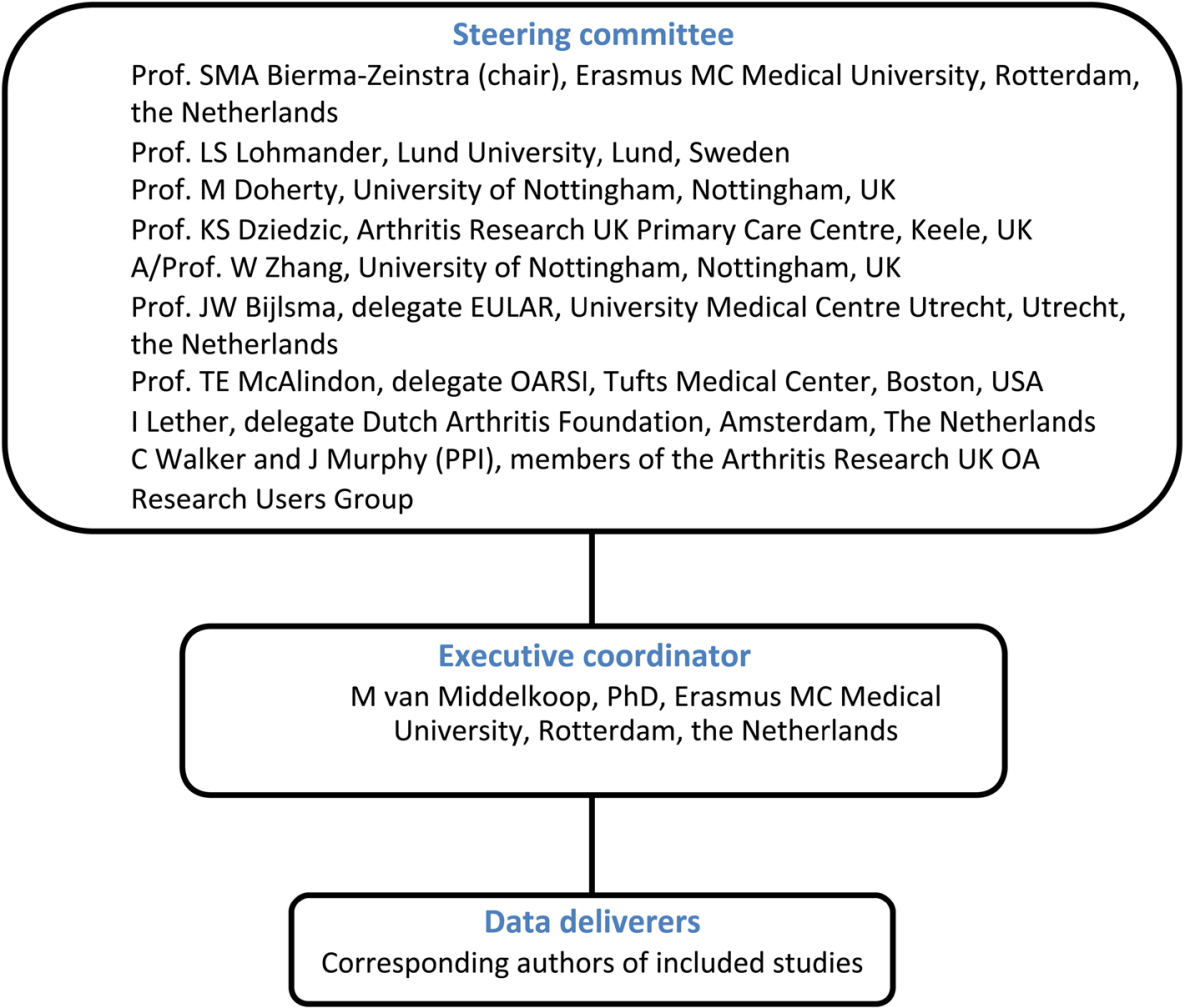
**Flowchart of the OA Trial Bank organization.** EULAR, European League Against Rheumatism; OARSI, OsteoArthritis Research Society International; PPI, patient and public involvement.

The described study protocol includes the first study of the OA Trial Bank, a worldwide collaboration that initiates meta-analyses on predefined subgroups of OA patients from existing literature. A chairman, steering committee and an executive coordinator formalize the OA Trial Bank (Figure 1). The steering committee guarantees continuity of the OA Trial Bank, supervises the executive coordinator and approves and agrees on all decisions made and methods applied (that is, agrees on the organizational structure and tasks of all parties involved; agrees on the design and methods of meta-analyses, definition of subgroups, statistical analyses), provides the content of the license agreement (data access, transfer and storage, predefined analyses, confidentiality and reliability statement, authorship) between data deliverers and the OA Trial Bank, and ensures handling of data and provides safe data storage. The steering committee currently consists of five internationally acknowledged clinical and epidemiological researchers in the OA field: a delegate of the main funder (Dutch Arthritis Foundation), a delegate of OsteoArthritis Research Society International, a delegate of the European League Against Rheumatism and two representatives of patient and public involvement (members of the Arthritis Research UK OA Research Users Group). The executive coordinator is responsible for the daily management of the OA Trial Bank, and performs or supervises the data analyses.

All publications arising from the OA Trial Bank will be made on behalf of the OA Trial Bank, and (co)authored (when applicable and following the Vancouver Protocol) by the data deliverers.

## Discussion

The described IPD meta-analysis is necessary to determine the efficacy of i.a. glucocorticoid injections in subgroups of hip and knee OA patients with severe pain and inflammatory signs. This is the first study that will combine the data of OA studies within the OA Trial Bank initiative in order to identify relevant subgroups for i.a. injection treatment using the IPD of the separate trials included in the proposed study.

One of the aims of this initiative is to build a solid and reliable organization for IPD analyses in OA trials; that is, the OA Trial Bank. The international character of the steering committee, the multidisciplinary structure, the patient involvement and the endorsement of OsteoArthritis Research Society International and the European League Against Rheumatism will all strengthen this initiative and hopefully inspire trialists (that is, data deliverers) to join this initiative. The steering committee ensures that rules of cooperation are registered and transparent towards data deliverers. Additionally, methods applied are all approved by the steering committee. This approach ensures a widely supported initiative improving the likelihood of successful data collection for existing trials.

It is intended that the OA Trial Bank initiative will coordinate future IPD analyses on all essential subgroup effects for the different intervention strategies applied in OA patients. The existing trial data on i.a. glucocorticoid injection will form an ideal starting point for our initiative: there are several recent publications on i.a. glucocorticoid injections, but it is not the most frequently studied intervention. With the experience gained in this first meta-analysis with IPD, the OA Trial Bank can pilot their methods and rules for data delivery and analysis in order to refine subsequent studies on OA subgroup effects for other frequently studied interventions.

All analyses performed with the IPD collected will be predefined. Since there are several dangers of observing spurious effects in any subgroup analysis, they should be predefined and interpreted cautiously. The numbers of patients included in meta-analyses performed with IPD afford greater statistical power and might therefore provide the only context in which it is actually reasonable to do subgroup analyses [19]. The IPD analysis, taking the interaction effects between subgroups and treatment into account, will therefore provide reliably information on subgroup effects that can be directly implemented in international guidelines on OA treatments.

Finally, the cooperation and publications of our initiative, apart from the primary outcomes, are likely to give direction towards other important variables measured – that is, for both confounders and subgroup variables – in future randomized trials. International consensus on variables measured in studies will make it easier for future meta-analyses with IPD to pool study data and adjust for potential confounders.

### Status of project

Currently, the OA Trial Bank has completed the search strategies in the different databases, has selected eligible trials for inclusion and is contacting the corresponding authors of the trials.

## Abbreviations

i.a: Intra-articular; IPD: Individual patient data; NSAID: Nonsteroidal anti-inflammatory drug; OA: Osteoarthritis.

## Competing interests

The authors declare that they have no competing interests.

## Authors’ contributions

All authors were involved in the study design and all will contribute to the interpretation of the results. MvM will contact the potential data deliverers, will coordinate the data collection, and performs or supervises the data analyses. MvM wrote the manuscript together with KD, MD, WZ, JWB, TM, SLSL, and SMAB-Z. MvM and SMAB-Z will have full access to the study data. All authors approved the final manuscript.

## Supplementary Material

Additional file 1 **Appendix 1.** Search strategy. Appendix 2. Criteria for a judgement of ‘yes’ for the sources of risk of bias. Click here for file
