# Supplementary material for: Individual patient data meta-analysis of trials investigating the effectiveness of intra-articular glucocorticoid injections in patients with knee or hip osteoarthritis: an OA Trial Bank protocol for a systematic review
Source: Syst Rev. 2013 Jul 5;2:54. doi: 10.1186/2046-4053-2-54 (PMC3707758; doi:10.1186/2046-4053-2-54)
Supplement: Additional file 1 — Appendix 1. Search strategy. Appendix 2. Criteria for a judgement of ‘yes’ for the sources of risk of bias. [file 2046-4053-2-54-S1.docx]

# ADDITIONAL FILES

Appendix 1. Search strategy

MEDLINE (PubMed)

((arthrit*[tw] OR arthros*[tw] OR arthrot*[tw] OR osteoarthro*[tw] OR osteoarthrit*[tw] OR osteoporo*[tw] OR boneloss*[tw] OR bone-loss*[tw] OR osteopen*[tw]) AND (hip[tw] OR hips[tw] OR knee[tw] OR knees[tw])) OR coxarthr*[tw] OR cox-arthr*[tw] OR gonarthr*[tw] OR gon-arthr*[tw]) AND (adrenal cortex hormones[mesh] OR adrenal cortex hormone*[tw] OR adrenal cortical hormone*[tw] OR adrenal cortical steroid*[tw] OR adrenal steroid*[tw] OR adrenocortical hormone*[tw] OR adrenocortical steroid*[tw] OR adrenocorticosteroid*[tw] OR adreno-corticosteroid*[tw] OR cortical steroid*[tw] OR cortico-steroid[tw] OR corticoid*[tw] OR corticosteroid*[tw] OR cortico-steroid*[tw] OR dermocorticosteroid*[tw] OR glucocortic*[tw] OR hydroxycorticosteroid*[tw] OR ketosteroid*[tw] OR androstenedion*[tw]) AND (intraartic*[tw] OR intra-artic*[tw]) AND inject*[tw] AND ((randomized controlled trial[pt] OR controlled clinical trial[pt] OR randomized controlled trials[mh] OR random allocation[mh] OR double-blind method[mh] OR single-blind method[mh] OR clinical trial[pt] OR clinical trials[mh] OR ("clinical trial"[tw]) OR ((singl*[tw] OR doubl*[tw] OR tripl*[tw]) AND (mask*[tw] OR blind*[tw])) OR ("latin square"[tw]) OR placebos[mh] OR placebo*[tw] OR random*[tw] OR research design[mh:noexp] OR comparative study[pt] OR evaluation studies[pt] OR follow-up studies[mh] OR prospective studies[mh] OR cross-over studies[mh] OR control[tw] OR controls[tw] OR controlled[tw] OR controled[tw] OR prospectiv*[tw] OR volunteer*[tw]) NOT (animals[mh] NOT humans[mh])) NOT (case report*[tw] OR retrospect*[tw] OR cadaver*[tw])

EMBASE

(((hip OR hips OR knee OR knees) NEAR/3 (arthrit* OR arthros* OR arthrot* OR osteoarthro* OR osteoarthrit*)) OR coxarthro* OR (cox NEAR/1 arthro*) OR gonarthro* OR (gon NEAR/1 arthro*)):ti,ab,de AND (corticosteroid/syn OR (adrenocorticosteroid* OR corticoid* OR corticosteroid* OR dermocorticosteroid* OR glucocortic* OR hydroxycorticosteroid* OR ketosteroid* OR androstenedion* OR (adrenal OR adrenocortical OR adreno OR cortical OR cortico) NEAR/3 (hormone* OR steroid* OR corticosteroid*)):ti,ab,de) AND ((intraartic* OR (intra NEAR/1 artic*)) AND inject*):ti,ab,de AND ((random*:ti,ab,de OR (clinical NEAR/1 trial*):ti,ab,de OR ((singl* OR doubl* OR tripl*) NEAR/3 (mask* OR blind*)):ti,ab,de OR 'latin square':ti,ab,de OR 'crossover procedure':ti,ab,de OR control*:ti,ab,de OR prospectiv*:ti,ab,de OR volunteer*:ti,ab,de) OR placebo*:ti,ab,de OR 'comparative study'/syn OR 'evaluation research'/syn OR 'follow up':ti,ab,de OR followup:ti,ab,de OR 'prospective study'/syn) NOT ([animals]/lim NOT [humans]/lim) NOT ((case NEAR/1 report*) OR retrospect* OR cadaver*):ti,ab,de

Web of Science

(((hip OR hips OR knee OR knees) NEAR/3 (arthrit* OR arthros* OR arthrot* OR osteoarthro* OR osteoarthrit*)) OR coxarthro* OR (cox NEAR/1 arthro*) OR gonarthro* OR (gon NEAR/1 arthro*)) AND (adrenocorticosteroid* OR corticoid* OR corticosteroid* OR dermocorticosteroid* OR glucocortic* OR hydroxycorticosteroid* OR ketosteroid* OR androstenedion* OR (adrenal OR adrenocortical OR adreno OR cortical OR cortico) NEAR/3 (hormone* OR steroid* OR corticosteroid*)) AND ((intraartic* OR (intra NEAR/1 artic*)) AND inject*) AND (randomi* OR ((singl* OR doubl* OR tripl*) NEAR/3 (mask* OR blind*)) OR "latin square" OR "crossover procedure" OR control* OR prospectiv* OR volunteer* OR placebo* OR "comparative study" OR "evaluation research" OR "follow up") NOT (animal* NOT human*) NOT ((case NEAR/1 report*) OR retrospect* OR cadaver*)

Cochrane Central

(((hip OR hips OR knee OR knees) NEAR/3 (arthrit* OR arthros* OR arthrot* OR osteoarthro* OR osteoarthrit*)) OR coxarthro* OR (cox NEAR/1 arthro*) OR gonarthro* OR (gon NEAR/1 arthro*)):ti,ab,kw AND (exp adrenal cortex hormones/ OR (adrenocorticosteroid* OR corticoid* OR corticosteroid* OR dermocorticosteroid* OR glucocortic* OR hydroxycorticosteroid* OR ketosteroid* OR androstenedion* OR ((adrenal OR adrenocortical OR adreno OR cortical OR cortico) NEAR/3 (hormone* OR steroid* OR corticosteroid*))):ti,ab,kw) AND ((intraartic* OR (intra NEAR/1 artic*)) AND inject*):ti,ab,kw

Cinahl

(((arthrit* OR arthros* OR arthrot* OR osteoarthro* OR osteoarthrit* OR osteoporo* OR boneloss* OR bone-loss* OR osteopen*) AND (hip OR hips OR knee OR knees)) OR coxarthr* OR cox-arthr* OR gonarthr* OR gon-arthr*) AND (adrenal cortex hormones OR adrenal cortex hormone* OR adrenal cortical hormone* OR adrenal cortical steroid* OR adrenal steroid* OR adrenocortical hormone* OR adrenocortical steroid* OR adrenocorticosteroid* OR adreno-corticosteroid* OR cortical steroid* OR cortico-steroid OR corticoid* OR corticosteroid* OR cortico-steroid* OR dermocorticosteroid* OR glucocortic* OR hydroxycorticosteroid* OR ketosteroid* OR androstenedion*) AND (intraartic* OR intra-artic*) AND inject* AND ((randomized controlled trial OR controlled clinical trial OR randomized controlled trials OR random allocation OR double-blind method OR single-blind method OR clinical trial OR clinical trials OR ("clinical trial") OR ((singl* OR doubl* OR tripl*) AND (mask* OR blind*)) OR ("latin square") OR placebos OR placebo* OR random* OR research design OR comparative study OR evaluation studies OR follow-up studies OR prospective studies OR cross-over studies OR control OR controls OR controlled OR controled OR prospectiv* OR volunteer*) NOT (animal* NOT human*)) NOT (case report* OR retrospect* OR cadaver*)

Scopus

TITLE-ABS-KEY(((hip OR hips OR knee OR knees) W/3 (arthrit* OR arthros* OR arthrot* OR osteoarthro* OR osteoarthrit*)) OR coxarthro* OR (cox W/1 arthro*) OR gonarthro* OR (gon W/1 arthro*)) AND TITLE-ABS-KEY(adrenocorticosteroid* OR corticoid* OR corticosteroid* OR dermocorticosteroid* OR glucocortic* OR hydroxycorticosteroid* OR ketosteroid* OR androstenedion* OR (adrenal OR adrenocortical OR adreno OR cortical OR cortico) W/3 (hormone* OR steroid* OR corticosteroid*)) AND TITLE-ABS-KEY((intraartic* OR (intra W/1 artic*)) AND inject*) AND TITLE-ABS-KEY(randomi* OR ((singl* OR doubl* OR tripl*) W/3 (mask* OR blind*)) OR "latin square" OR "crossover procedure" OR control* OR prospectiv* OR volunteer* OR placebo* OR "comparative study" OR "evaluation research" OR "follow up") AND NOT TITLE-ABS-KEY(animal* AND NOT human*) AND NOT TITLE-ABS-KEY((case W/1 report*) OR retrospect* OR cadaver*)

Appendix 2.Criteria for a judgement of ‘yes’ for the sources of risk of bias

*1. Was the method of randomization adequate?*

A random (unpredictable) assignment sequence. Examples of adequate methods are coin toss (for studies with two groups), rolling a dice (for studies with two or more groups), drawing of balls of different colours, drawing of ballots with the study group labels from a dark bag, computer-generated random sequence, pre-ordered sealed envelops, sequentially-ordered vials, telephone call to a central office, and pre-ordered list of treatment assignments. Examples of inadequate methods are: alternation, birth date, social insurance/security number, date in which they are invited to participate in the study, and hospital registration number

*2. Was the treatment allocation concealed?*

Assignment generated by an independent person not responsible for determining the eligibility of the patients. This person has no information about the persons included in the trial and has no influence on the assignment sequence or on the decision about eligibility of the patient.

Was knowledge of the allocated interventions adequately prevented during the study?

*3. Was the patient blinded to the intervention?*

This item should be scored “yes” if the index and control groups are indistinguishable for the patients or if the success of blinding was tested among the patients and it was successful.

*4. Was the care provider blinded to the intervention?*

This item should be scored “yes” if the index and control groups are indistinguishable for the care providers or if the success of blinding was tested among the care providers and it was successful.

*5. Was the outcome assessor blinded to the intervention?*

Adequacy of blinding should be assessed for the primary outcomes. This item should be scored “yes” if the success of blinding was tested among the outcome assessors and it was successful or: for patient-reported outcomes in which the patient is the outcome assessor (e.g., pain, disability): the blinding procedure is adequate for outcome assessors if participant blinding is scored “yes” for outcome criteria assessed during scheduled visit and that supposes a contact between participants and outcome assessors (e.g., clinical examination): the blinding procedure is adequate if patients are blinded, and the treatment or adverse effects of the treatment cannot be noticed during clinical examination for outcome criteria that do not suppose a contact with participants (e.g., radiography, magnetic resonance imaging): the blinding procedure is adequate if the treatment or adverse effects of the treatment cannot be noticed when assessing the main outcome for outcome criteria that are clinical or therapeutic events that will be determined by the interaction between patients and care providers (e.g., co-interventions, hospitalization length, treatment failure), in which the care provider is the outcome assessor: the blinding procedure is adequate for outcome assessors if item “E” is scored “yes” for outcome criteria that are assessed from data of the medical forms: the blinding procedure is adequate if the treatment or adverse effects of the treatment cannot be noticed on the extracted data.

Were incomplete outcome data adequately addressed?

*6. Was the drop-out rate described and acceptable?*

The number of participants who were included in the study but did not complete the observation period or were not included in the analysis must be described and reasons given. If the percentage of withdrawals and drop-outs does not exceed 20% for during follow-up and does not lead to substantial bias a 'yes' is scored. (N.B. these percentages are arbitrary, not supported by literature).

*7. Were all randomized participants analysed in the group to which they were allocated?*

All randomized patients are reported/analyzed in the group they were allocated to by randomization for the most important moments of effect measurement (minus missing values) irrespective of non-compliance and co-interventions.

Other sources of potential bias:

*8. Were the groups similar at baseline regarding the most important prognostic indicators?*

In order to receive a “yes”, groups have to be similar at baseline regarding demographic factors, severity of complaints, and value of main outcome measure(s).

*9. Were co-interventions avoided or similar?*

This item should be scored “yes” if there were no co-interventions or they were similar between the index and control groups.

*10. Was the compliance acceptable in all groups?*

The reviewer determines if the compliance with the interventions is acceptable, based on the reported intensity, duration, number and frequency of sessions for both the index intervention and control intervention(s). For example, physiotherapy treatment is usually administered over several sessions; therefore it is necessary to assess how many sessions each patient attended. For single-session interventions (for ex: surgery), this item is irrelevant.

*11. Was the timing of the outcome assessment similar in all groups?*

Timing of outcome assessment should be identical for all intervention groups and for all important outcome assessments.

Note: These instructions are adapted from van Tulder 2003, Boutron et al, 2005 (CLEAR NPT) and the Cochrane Handbook of Systematic Reviews of Interventions.
